# Evaluation of Task Scheduling Algorithms in Heterogeneous Computing Environments

**DOI:** 10.3390/s21175906

**Published:** 2021-09-02

**Authors:** Roxana-Gabriela Stan, Lidia Băjenaru, Cătălin Negru, Florin Pop

**Affiliations:** 1 Computer Science and Engineering Department, University Politehnica of Bucharest (UPB), 060042 Bucharest, Romania; roxana_gabriela.stan@upb.ro (R.-G.S.); lidia.bajenaru@upb.ro (L.B.); catalin.negru@upb.ro (C.N.); 2 National Institute for Research and Development in Informatics (ICI), 011455 Bucharest, Romania

**Keywords:** heterogeneous computing, hybrid edge–cloud environments, task scheduling, performance evaluation framework

## Abstract

This work establishes a set of methodologies to evaluate the performance of any task scheduling policy in heterogeneous computing contexts. We formally state a scheduling model for hybrid edge–cloud computing ecosystems and conduct simulation-based experiments on large workloads. In addition to the conventional cloud datacenters, we consider edge datacenters comprising smartphone and Raspberry Pi edge devices, which are battery powered. We define realistic capacities of the computational resources. Once a schedule is found, the various task demands can or cannot be fulfilled by the resource capacities. We build a scheduling and evaluation framework and measure typical scheduling metrics such as mean waiting time, mean turnaround time, makespan, throughput on the Round-Robin, Shortest Job First, Min-Min and Max-Min scheduling schemes. Our analysis and results show that the state-of-the-art independent task scheduling algorithms suffer from performance degradation in terms of significant task failures and nonoptimal resource utilization of datacenters in heterogeneous edge–cloud mediums in comparison to cloud-only mediums. In particular, for large sets of tasks, due to low battery or limited memory, more than 25% of tasks fail to execute for each scheduling scheme.

## 1. Introduction

Scheduling of execution workloads is critical to the performance of any computing system. In particular, large-scale processing of heterogeneous big data in heterogeneous computing environments is prone to suffer from high latency due to factors such as nonoptimal job scheduling and resource allocation, as well as Internet of Things (IoT) mobility. Considering the unprecedented amount of smart edge devices and IoT sensors currently, massive volumes of heterogeneous data from a variety of use cases are generated continuously [1]. This directly translates into an emerging need for latency-aware, energy-efficient computing techniques [2]. There is a wide research interest in formulating optimized scheduling strategies addressing the above considerations [3,4,5]. To test the performance of current techniques, it is important to have a framework for their accurate and correct evaluation.

By conducting an extensive literature review of current advances in the field of task scheduling on heterogeneous distributed systems [6,7], we identified a pressing need for more clarity and formalism in the performance assessment of existing scheduling algorithms such as Min-Min and Max-Min, introduced in [8].

The problem we address in this paper is the evaluation of the scheduling algorithms Round-Robin (RR), Shortest Job First (SJF), Min-Min, and Max-Min for heterogeneous environments. These algorithms have been extensively studied in the scheduling literature [9,10], and we think they are appropriate choices to evaluate and decide whether they can be good candidates for heterogeneous computing. This work can be expanded in the future to analyze other scheduling algorithms (e.g., fair and capacity scheduling, resource-aware scheduling, genetic algorithm).

We clearly define the cloud and edge resource capacities such as the CPU and memory. To achieve a realistic setup, we further define the heterogeneity of the execution workload as a combination of read and write tasks with different CPU, memory, and I/O requirements.

The results we obtained give a novel analysis of the previously mentioned algorithms taking into account that certain resource capacities of edge devices may be insufficient to execute a task. Our analysis clarifies when the existing scheduling algorithms can be safely used and by how much their performance is affected when assigning tasks to execute on low-capacity resources.

Finally, we identify the need to develop further scheduling algorithms that take into consideration the above computational constraints. This is the subject of future work.

### 1.1. Contributions

The research contributions of this paper are the following:

- Mathematically defining the scheduling model for heterogeneous computing, which is used in our performance evaluations;
- Building a framework (https://github.com/roxana-stan/2HD-Scheduling-Algorithms-Evaluation-Framework—accessed on 15 August 2021) for the evaluation of scheduling algorithms on edge–cloud heterogeneous environments. Furthermore, as part of the framework, we implemented a comprehensive set of performance evaluation metrics;
- Evaluating the performance of state-of-the-art scheduling algorithms against the set of metrics by conducting experiments through our framework. We chose heterogeneous tasks with representative demands and different capacities of computing resources;
- Assessing the performance degradation in terms of the task failures and nonoptimal resource utilization of conventional scheduling algorithms. This is key to our conclusions that future scheduling algorithms need to be developed in order to better optimize execution for low-capacity edge resources in the context of an edge–cloud setup.

### 1.2. Structure

We present a brief outline of the paper. Section 2 gives an overview of the task scheduling procedure, highlighting various objectives and detailing the steps of the scheduling process in a cooperative edge–cloud system. In Section 3, we theoretically define our proposed scheduling model for heterogeneous computing environments. Section 4 explains the concept of heterogeneous workloads and lists the possible types of tasks. In Section 5, we establish an evaluation methodology based on a complete set of metrics, highlighting the parameters for realistic experiments, the assumptions, and the configuration of diverse resources. Section 6 covers the performance analysis of different scheduling algorithms. We discuss the experimental results, and in Section 7, we summarize the conclusions of this research work.

## 2. Task Scheduling

Our paper evaluates several techniques that address the independent task scheduling problem, also known as Bag-of-Tasks (BoT) workload scheduling [11]. Given an application comprised of tasks with no interdependence among them, the scheduling challenge consists of assigning each task the most suitable computation resource for its timely execution. However, the proposed evaluation methodology can be applied to workflow scheduling as well in the case of tasks with precedence constraints [12].

Generally, the problem of optimally scheduling tasks on computing machines has been proven to be NP-complete [13], hence the necessity to rely on heuristics.

In a distributed computing environment, the challenge is whether to allocate edge resources or cloud resources to the incoming tasks. In particular, in this paper, we consider edge datacenters to be equipped with battery-powered edge devices such as smartphones and Raspberry Pis.

By processing the tasks within edge datacenters, we benefit from reduced energy utilization in comparison to excessive consumption in traditional cloud datacenters. The drawback of edge datacenters is that they have less processing power than cloud datacenters [14], and hence, the scheduling strategy is vital for tasks with highly variable sizes.

### 2.1. Scheduling Objectives

We distinguish several functions a scheduling strategy aims to accomplish:

- Achieve a high system throughput;
- Minimize the response time of the system;
- Minimize the overall completion time of all tasks. Optionally, fulfil the workload’s deadline, if any;
- Minimize the cost of the edge–cloud computing services;
- Minimize the energy consumption of the datacenters;
- Efficiently utilize the system’s resources.

### 2.2. Scheduling Steps

**Figure 1:**
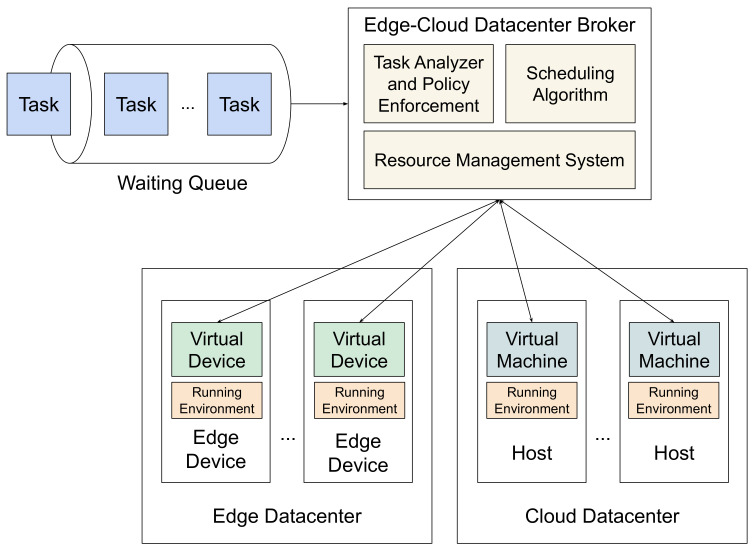
Scheduling in an edge–cloud collaborative computing environment.

The architecture of the scheduling process is given in Figure 1. In this framework, an edge–cloud datacenter broker is responsible for workload scheduling, and a single queue holds all ready tasks to be submitted for execution.

The steps of the scheduling process are as follows:

1. The broker discovers and filters the set of available Virtual Machine (VM) and Virtual Device (VD) resources by inspecting their statuses;
2. The scheduling algorithm computes the suitable processing order of tasks;
3. Once the algorithm completes and the distribution of tasks is decided, the broker submits the tasks to the elected computing resources.

## 3. Scheduling Model for Heterogeneous Computing

In this section, we propose and formally state a model for independent task scheduling in hybrid computing environments.

### 3.1. Task Scheduling Problem

We start by formulating mathematically the task scheduling problem.

A heterogeneous computing system comprises cloud and edge datacenters. Furthermore, a datacenter DC consists of a set of physical machines, either hosts or edge devices: $\left\{PM_{1},PM_{2},\ldots ,PM_{m}\right\}$. The system’s datacenters provide a set *R* of cloud and edge resources: $\left\{R_{1},R_{2},\ldots ,R_{r}\right\}$ in the form of virtual machines or virtual devices.

We define the set of *t* independent tasks: $\left\{T_{1},T_{2},\ldots ,T_{t}\right\}$ as the workload W. In our setting, we consider t>>r.

Each task $T_{i}$ has a number of instructions $instr_{i}$ and associated data, which have to be uploaded before or downloaded after execution. We denote the upload data size by $upload_{i}$ and the download data size by $download_{i}$. We say that the task $T_{i}$ is characterized by the triplet:

(1) $$T_{i}=\left(upload_{i},instr_{i},download_{i}\right).$$

Each processing resource $R_{j}$ has specific capacities, namely its computing power $cpu_{j}$, defined in million instructions per second (MI/s), and memory $mem_{j}$ and bandwidth $bw_{j}$ capacities:

(2) $$R_{j}=\left(cpu_{j},mem_{j},bw_{j}\right).$$

We define the expected execution time $ET_{i,j}$ of a task $T_{i}$ on a resource $R_{j}$ as the sum of the data transfer times and the task’s computation time:

(3) $$ET_{i,j}=\frac{upload_{i}}{bw_{j}}+\frac{instr_{i}}{cpu_{j}}+\frac{download_{i}}{bw_{j}}.$$

Furthermore, we define the expected completion time $CT_{i,j}$ of the task $T_{i}$. This quantity represents the time at which the task has completed processing and exits the system. Thus, $CT_{i,j}$ is the sum of the task’s arrival time $AT_{i}$, waiting time $WT_{i}$, and execution time on the resource $R_{j}$:

(4) $$CT_{i,j}=AT_{i}+WT_{i}+ET_{i,j}.$$

We mathematically formulate the task scheduling problem as finding a scheduling sequence *S* in which each task is assigned a resource for its execution:

(5) $$S=\{\left(T_{i},R_{j}\right)|\forall T_{i}\in W,\exists R_{j}\in R\;s.t.\;T_{i}\;\mathrm{is}\;\mathrm{scheduled}\;\mathrm{on}\;R_{j}\}.$$

### 3.2. Resource Utilization

Having defined the scheduling problem, it remains to mathematically define the concept of resource utilization that we use throughout the paper.

The capacity of a host or edge device $PM_{k}$ is characterized by the CPU capacity ${PM_{k}}_{cpu}$, the memory capacity ${PM_{k}}_{mem}$, and the I/O capacity ${PM_{k}}_{io}$:

(6) $$PM_{k}\;capacity:=\left(PM_{kcpu},PM_{kmem},PM_{kio}\right).$$

We define the tuple $R_{j}\;load$:

(7) $$R_{j}\;load:=\left({R_{j}}_{cpu},{R_{j}}_{mem},{R_{j}}_{io}\right).$$

It characterizes the CPU load ${R_{j}}_{cpu}$, memory load ${R_{j}}_{mem}$, and I/O load ${R_{j}}_{io}$ of a resource $R_{j}$.

We now define the resource utilization $RU_{k}$ of each datacenter machine $PM_{k}$ in terms of the CPU, memory, and I/O utilization at time *t*:

(8) $$CPU\;utilization:={RU_{k}}_{cpu}\left(t\right)=\frac{\sum_{j}a_{j}\left(t\right)\cdot {R_{j}}_{cpu}}{PM_{kcpu}}$$

(9) $$Memory\;utilization:={RU_{k}}_{mem}\left(t\right)=\frac{\sum_{j}a_{j}\left(t\right)\cdot {R_{j}}_{mem}}{PM_{kmem}}$$

(10) $$I/O\;utilization:={RU_{k}}_{io}\left(t\right)=\frac{\sum_{j}a_{j}\left(t\right)\cdot {R_{j}}_{io}}{PM_{kio}}$$

for all $R_{j}$ hosted by $PM_{k}$, where $a_{j}\left(t\right)=1$, if $R_{j}$ is loaded at time *t*, and 0 otherwise.

We average the above three utilization measurements to define the resource utilization $RU_{k}$ at time *t* as such:

(11) $$RU_{k}\left(t\right)=\frac{{RU_{k}}_{cpu}\left(t\right)+{RU_{k}}_{mem}\left(t\right)+{RU_{k}}_{io}\left(t\right)}{3}.$$

Finally, we define the resource utilization $RU_{DC}$ of the datacenter DC. This value totals all datacenter machines’ resource utilization $RU_{k}\left(t\right)$, starting from the first task’s arrival time t=a to the last task’s completion time t=c. Mathematically, we represent $RU_{DC}$ as the following integral:

(12) $$RU_{DC}=\int_{a}^{c}\frac{\sum_{k=1}^{m}RU_{k}\left(t\right)}{m}\;dt.$$

For simplicity, we assume ${R_{j}}_{cpu}=cpu_{j}$, ${R_{j}}_{mem}=mem_{j}$, and ${R_{j}}_{io}=bw_{j}$ at any time resource $R_{j}$ is loaded.

## 4. Task Types and Heterogeneous Workloads

Heterogeneous execution workloads are decomposed into different fixed-size tasks. We distinguish between two classes of tasks, namely read and write tasks, depending on the type of the atomic operations they perform.

**Read Task**

**Figure 2:**
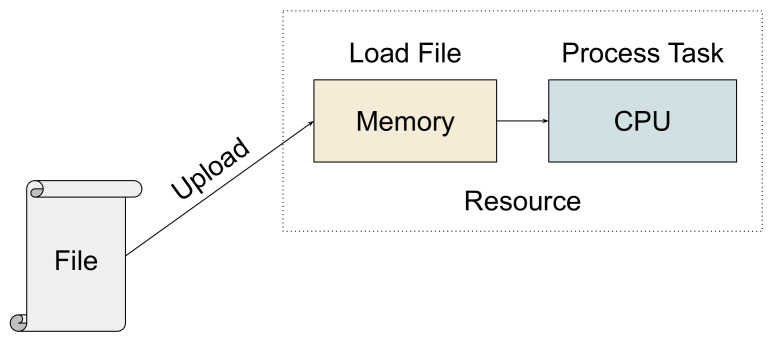
Execution flow of a read task on a computational resource.

As illustrated in Figure 2, the execution of a read task on a resource consists of the task’s file upload operation, the loading of the input data into memory, followed by the actual CPU processing of the task.

Thus, in addition to the computational demand, a read task necessitates a certain memory capacity of the resource. The size of the data to be read into memory has a practical implication on the ability to process the task on an edge resource with constrained memory.

**Write Task**

**Figure 3:**
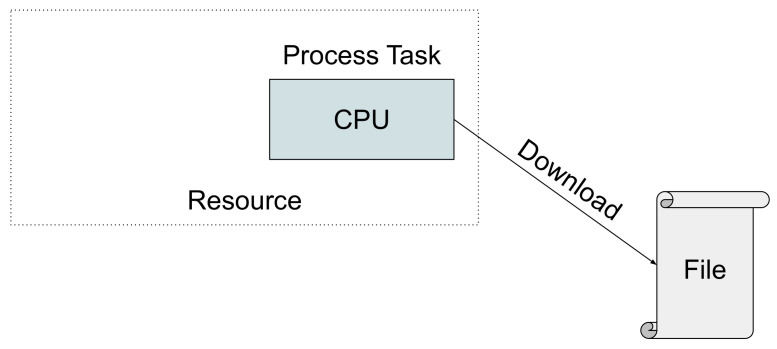
Execution flow of a write task on a computational resource.

The execution of a write task on a resource involves a processing and a download operation, thereby writing the task’s output data directly to a file, as illustrated in Figure 3.

We assume write tasks operate on a negligible amount of memory, so there are no limitations of the edge resources regarding their ability to execute such tasks.

### 4.1. Task Types

We introduce different types of read and write tasks, of varying lengths and data sizes. In this way, we aim to cover any possible combination of computation and storage requirements. Table 1 and Table 2 illustrate the characteristics of the proposed tasks and their heterogeneous processing demands. The listed CPU requirements are based on the assumption that the associated task has a tractable execution time.

The following are examples of how each read task can be used in practice:

- RT1 can represent financial modeling based on a large historical dataset stored in-memory;
- RT2 can represent the computation of an NP-hard optimization problem based on a small input dataset;
- RT3 can represent light database queries performed on a large dataset stored in-memory;
- RT4 can represent light video editing.

### 4.2. Heterogeneous Workloads

We model any heterogeneous workload as a set of read and write tasks.

Denote $r_{1}$, $r_{2}$, $r_{3}$, and $r_{4}$ the number of tasks of type RT1, RT2, RT3, and RT4, respectively. We define the total number of read tasks, *r*, given by:

(13) $$r_{1}+r_{2}+r_{3}+r_{4}=r.$$

Similarly, let $w_{1}$, $w_{2}$, $w_{3}$, and $w_{4}$ be the number of tasks of type WT1, WT2, WT3, and WT4, and the total number of write tasks, *w*:

(14) $$w_{1}+w_{2}+w_{3}+w_{4}=w.$$

Considering this, a heterogeneous workload consisting of *t* tasks has f·t read tasks and (1−f)·t write tasks, where f∈[0,1] represents the heterogeneity factor. Depending on the value of *f*, we distinguish between the following types of workloads:

- Read-only workloads: f=1;
- Write-only workloads: f=0;
- Read-write workloads: f∈(0,1).

## 5. Performance Evaluation Methodology

This section details the setup for our performance analysis of different scheduling algorithms in hybrid computing systems.

Our study in heterogeneous edge–cloud environments was simulation-based. We conducted extensive experiments by employing a realistic setting of the IoTSim-Edge simulator [15], which extends the cloud computing capabilities of the CloudSim framework [16] with edge processing. IoTSim-Edge enables us to model distinctive features of an edge computing environment, such as the battery consumption of the edge devices. It can also model dynamic IoT environments with different types of IoT sensors and edge devices in line with various real-life use cases.

On top of the IoTSim-Edge simulation tool set, we added our 2HD evaluation framework (https://github.com/roxana-stan/2HD-Scheduling-Algorithms-Evaluation-Framework, accessed on 15 August 2021) of scheduling algorithms in hybrid edge–cloud computing environments by providing support for performance metrics computation, implementing the scheduling policies, and configuring the parameters of each experiment.

### 5.1. Resource Configurations

Table 3 contains the configurations of the heterogeneous resources we considered for our experiments in hybrid edge–cloud environments. The chosen resources possess different computation, system memory, and network bandwidth capacities. As the reader may notice, whereas there are no processing limitations of cloud resources, edge resources have limited capacities for processing the tasks given in Table 1 and Table 2. The bandwidth values were adjusted to accommodate key network attributes such as the communication medium and the data transmission distance.

We chose the memory capacities for each machine based on the specifications of Raspberry Pi 3 Model B [17], Samsung Galaxy S9 [18], and Mac Pro 2019 [19]. Experiments can be easily reevaluated using different device specifications.

According to the proposed theoretical model and based on the resources’ configurations (Table 3) and tasks’ requirements (Table 1 and Table 2), we record in Table 4 the expected execution times of each task type on each resource type. For instance, the total time spent on executing a task RT2 on a resource R was 90 s, representing the sum of the data transfer time (40 s = 0.2 × 1000 MB/5 MB/s) and the processing time (50 s = 4,000,000 MI/80,000 MI/s).

The read tasks RT1 and RT3 given in Table 1 have requirements that cannot be fulfilled by the edge resources due to their restricted memory capacities for performing the tasks’ necessary operations.

We assumed that the edge devices are battery powered to conduct energy consumption evaluations. However, the reader should bear in mind that in other contexts, edge devices can be connected to the power grid [20]. Table 5 contains several battery performance values we considered depending on the edge device. This translates to the fact that the battery of a smartphone lasts 5 h for processing or 8 h for data transfer, and for a Raspberry Pi, the battery drains after 3 h of processing or 5 h of data transfer.

### 5.2. Technical Assumptions

We modeled the sequence of the tasks entering the heterogeneous system as a Poisson arrival process of rate λ. The rate λ measures the average number of the incoming tasks per unit of time. The tasks are passed to each scheduling policy in ascending order of their arrival times.

We enabled the creation of exactly one resource within a physical machine by matching their characteristics: CPU rating, number of CPUs, memory, and bandwidth. In addition, we created datacenters that contained only physical machines of the same type.

We formulated the following assumptions on the task scheduling routine:

- Each task requires exactly one computing resource and cannot be migrated to other resources once its execution starts. The task cannot be preempted;
- There are no variations of the available resources: their configurations are not modified over time, and no other resources are added or removed later.

The assumptions regarding the heterogeneous computing environment include:

- The edge devices do not have the mobility feature enabled;
- We relied on a stable Internet connection for our distributed system and assumed that all datacenters and their attached resources are available for task processing;
- We fixed the following order of the computing resources: smartphone, Raspberry Pi, and cloud, to mimic their geographical proximity.

### 5.3. Algorithms

For our analysis, we considered the following four bag-of-tasks scheduling algorithms. A succinct description of these techniques follows.

#### 5.3.1. Round-Robin

In this scheme, tasks are allocated resources in a circular manner. Consequently, all resources are considered equal regardless of their processing power. The aim of this policy for homogeneous contexts is to equalize the tasks’ load on the resources [21].

#### 5.3.2. Shortest Job First

This scheme sorts tasks in increasing order of their execution times and allocates each task a resource using the RR scheme. As we were facing the challenge of resource heterogeneity, we defined the execution time of a task as the weighted average value of its execution times for each resource type.

#### 5.3.3. Min-Min and Max-Min

The algorithms D and E in [8] are known as the Min-Min and Max-Min algorithms [22]. In comparison to the Round-Robin and SJF methods, the Min-Min and Max-Min algorithms make the decision of assigning a task to a computing resource by considering the task’s execution on each resource.

Both scheduling policies perform the following steps in a recursive fashion: find each task’s minimum completion time among the resources, then select the task having the minimum, respectively the maximum, completion time among all yet unassigned tasks. The broker submits the task to the resource, which obtains that completion time, and repeats the process.

As a noticeable drawback, the algorithms’ formula for the task’s completion time does not take into consideration the task’s arrival time.

Further analysis can be also made on the algorithms proposed in the microservice platforms, such as fair and capacity scheduling [23]. However, given their underlying policy of the type First-In First-Out (FIFO), we expect these schedulers to show similar performances.

### 5.4. Metrics

We measured a comprehensive set of metrics, which enabled us to evaluate the scheduling algorithms and to assess the performance of a computing system as well [21]. Each metric was implemented by us and is publicly available as part of our evaluation framework.

We refer to the following definitions, commonly used in the task scheduling literature:

- *Arrival time*: the specific time when the task enters the system and is ready for execution;
- *Completion time*: the specific time when the task’s execution completes and the task exits the system;
- *Execution time*: the required processing time to execute a task on a computation resource;
- *Turnaround or response time*: the total time spent by the task in the system, from the task’s arrival until its completion:
  (15) Turnaround time=Completion time−Arrival time;
- *Waiting or queueing time*: the amount of time the task is ready for execution and waits in the system:
  (16) Waiting time=Turnaround time−Execution time;
- *Makespan or schedule length*: the overall time taken to execute all tasks, from the tasks’ arrival until their completion;
- *Throughput*: the rate of task completions in the computing system per time unit;
- *Resource utilization*: the fraction of the tasks’ overall execution time the datacenter’s resources are utilized.

### 5.5. Experimental Setup

We note the following simulation instructions guiding our experiments:

- Set the tasks’ average arrival rate λ to 0.8 (tasks per second). In other words, in 100 s, we expect 80 tasks to arrive and be added to the waiting queue;
- Have a volume of tasks to be processed higher than the number of available resources;
- Each task is executed on one CPU. This does not limit the scope of our results, but simplifies the implementation;
- Configure each datacenter machine, either the host or edge device, with one CPU unit;
- Create one broker instance, implementing a specific scheduling algorithm;
- Create one cloud datacenter and two edge datacenters, holding a list of hosts, respectively edge devices of the smartphone and Raspberry Pi types.

We conducted realistic experiments given the parameters in Table 6, by varying the number of tasks: we evaluated the scheduling algorithms on 2000 and 8000 tasks. In homogeneous contexts, these execution workload capacities translate on average to 100 tasks per resource for the first experiment and 400 tasks per resource for the second one. To compare the scheduling algorithms within an experiment, we ran each algorithm on the same set of tasks ensuring the same task arrival order.

In our experiments, we used equal fractions of read and write task types: workload heterogeneity factor f=0.5 and r1=r2=r3=r4=0.25, respectively w1=w2=w3=w4=0.25. In future works, we plan to vary the tasks’ distribution by employing specific workload profiles for cloud and edge datacenters.

## 6. Experimental Results

Using the evaluation setup detailed in the previous section, we discuss the obtained experimental results, detailing the performance of the considered algorithms.

### 6.1. Cloud-Only and Edge–Cloud Experimentation Testbeds

We first implemented the scheduling algorithms and collected the metric measures for the scenario in which only the cloud resources hosted within the cloud datacenter are available. Afterwards, we created the edge datacenters and added the edge resources such that the scheduling methods can utilize the entire set of edge–cloud resources.

The differences in the recorded measurements between the cloud-only and edge–cloud setups are as follows.

In the first experiment, both setups gave comparable makespan values when using either RR or SJF policies. However, if Min-Min or Max-Min policies were used, the makespan values were improved in the edge–cloud setup. When 8000 tasks were executed, we obtained that RR and SJF policies also delivered lower makespan values in the edge–cloud setup, at the cost of a large percentage of tasks failing (see Section 6.3 for more details). A very similar behavior of the algorithms applies to the system’s throughput measure.

As expected, the scheduling schemes reported higher turnaround and waiting times when considering only cloud resources.

Generally speaking, the algorithms showed noticeable improvements in the edge–cloud ecosystem as we progressively increased the number of tasks. For scenarios with small sets of tasks (e.g., 80, 800), we experienced inconsistent results, and an edge–cloud setup did not make sense entirely. We are interested in handling high volumes of tasks, and our simulations confirmed that employing an edge–cloud environment for large workloads is preferred to a cloud-only setup, which delivered poor performance (low throughput, infeasible waiting and response times).

**Unavailable Resources**

By introducing edge devices that are battery powered, the policies failed to schedule several tasks. This was due to the fact that certain edge resources became unavailable once their corresponding edge devices reached a low level of the battery, below 20% of its capacity. More generally, the conventional scheduling schemes do not inspect the status of the resources.

**Limited Resource Capacity**

A task failure can also occur if the task has a requirement that cannot be satisfied by the assigned resource due to its restricted capacity. Specifically, if read tasks RT1 and RT3 are allocated to edge resources, these tasks fail to execute.

Within the scheduling framework, the broker has the responsibility to evaluate the previous cases: unavailable resources and limited resource capacity. If any of the tasks are scheduled to ineligible resources by the scheduling algorithm, they are marked as failed and are dropped from the scheduling process. Thus, the broker does not force the reassignment of these tasks to suitable resources.

### 6.2. Performance Degradation in Heterogeneous Contexts

We further review the efficiency of the state-of-the-art scheduling algorithms in hybrid edge–cloud computing environments. Table 7, Table 8 and Table 9 collect the measurements of the specific scheduling metrics, namely: mean turnaround time, mean waiting time, schedule length, and system throughput.

In edge–cloud contexts, the recorded measures of each metric vary greatly across algorithms, in contrast to cloud-only environments, where the values are comparable.

Table 7 displays no major differences in the average waiting and turnaround times of the scheduling policies per experiment, showing slight improvements for Min-Min and Max-Min.

As illustrated in Table 8 and Table 9, the Min-Min and Max-Min algorithms gave substantial performance boosts in terms of makespan and throughput. Thus, in heterogeneous contexts, a policy that takes into consideration the tasks’ processing speed on each resource is vital for attaining the desired performance.

Overall, the SJF heuristic proved to be inefficient in edge–cloud environments, despite the fact that we adapted the task execution time computations for heterogeneous resources. Even though SJF is said to provide the minimum average waiting time among all scheduling policies in cloud systems [24], our simulations showed that this statement does not apply to heterogeneous mediums.

Finally, the results showed that none of the scheduling algorithms gave high throughput outcomes. Thus, there needs to be future work that optimizes this value and gives high system performance in heterogeneous mediums.

### 6.3. Failed Tasks

**Figure 4:**
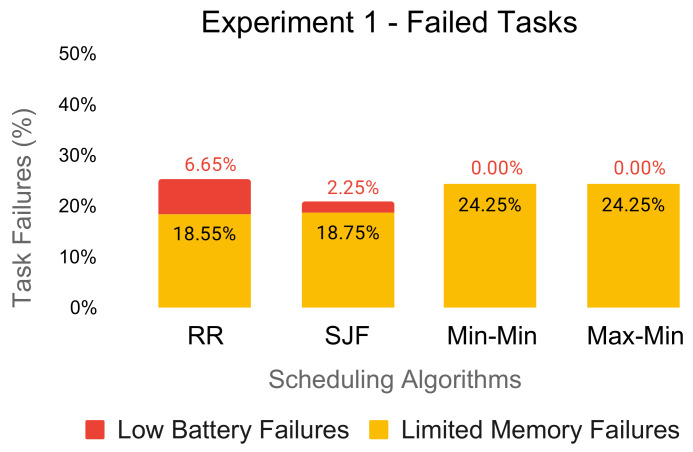
Percentage of failed tasks for Experiment 1 (2000 tasks).

**Figure 5:**
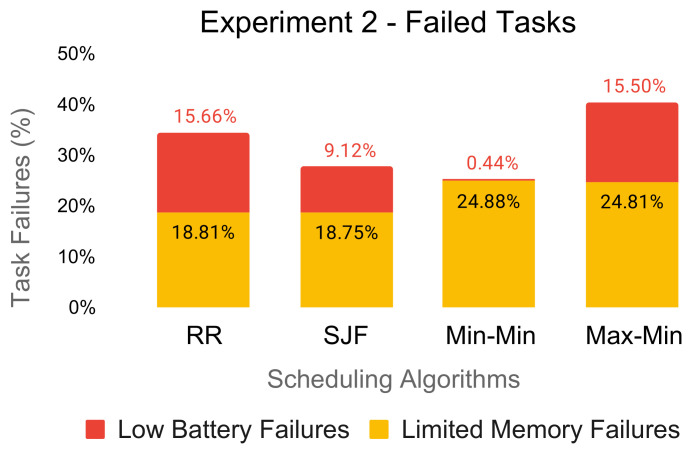
Percentage of failed tasks for Experiment 2 (8000 tasks).

In Figure 4 and Figure 5, we illustrate the percentage of dropped tasks due to the limited memory capacity of the edge resources and the low battery of the underlying edge devices. For each experiment, the amount of task failures varied considerably across the scheduling algorithms.

The Round-Robin and SJF schedules produced low-battery failures even for the first experiment of 2000 tasks. Low-battery failures continued to increase when we processed more tasks on the edge resources as the batteries of the underlying edge devices continued to drain, as seen in Figure 5.

In terms of the limited memory failures, Min-Min and Max-Min performed poorly. Specifically, the algorithms do not consider the memory capacity restriction of the edge resources, selecting each time the task obtaining the minimum completion time among all of the available resources.

Both experiments showed per algorithm very similar percentages of limited memory failures regardless of the number of tasks to be scheduled. We were able to reproduce this behavior even on experiments with less demanding workloads (e.g., 800 tasks). Therefore, we can state that this performance issue is not dependent on the amount of computational resources available in the system.

Considering that any evaluated scheduling technique results in large quantities of dropped tasks, it is obvious that the traditional algorithms fail to find valid schedules of the execution workloads. Therefore, it is mandatory to develop hybrid implementations of these algorithms for their proper functioning in a collaborative edge–cloud system.

### 6.4. Nonoptimal Resource Utilization

**Figure 6:**
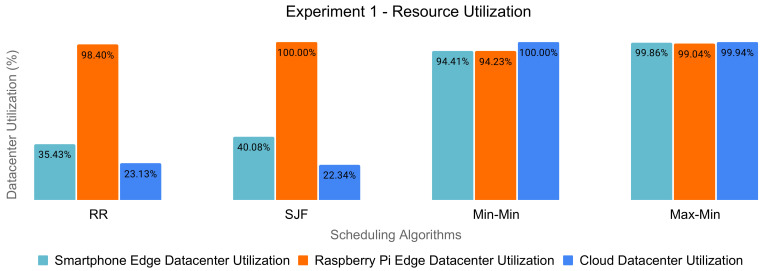
Datacenters’ resource utilization for Experiment 1 (2000 tasks).

**Figure 7:**
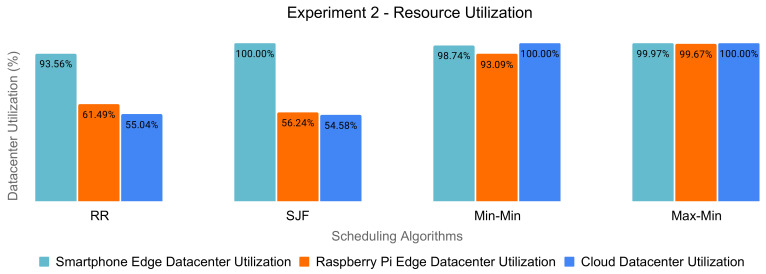
Datacenters’ resource utilization for Experiment 2 (8000 tasks).

For each experiment, we examined the edge and cloud datacenters’ resource utilization of the scheduling policies, according to Figure 6 and Figure 7.

We observed a clear imbalance in utilizing the resources in the case of Round-Robin and SJF. On the other hand, the Min-Min and Max-Min algorithms obtained a heavy load of the machines of each datacenter. However, judging by the fact that there was a high percentage of dropped tasks, their reassignment and execution on eligible resources would lead to low effectiveness in resource usage.

Therefore, the traditional scheduling policies do not target the optimal utilization of the resources in order to maximize the energy efficiency of the edge and cloud datacenters.

Even though Max-Min suffered from the highest percentage of failed tasks, Max-Min was the better performer out of all tested scheduling algorithms across all experiments. However, a preference to either opt for Min-Min or Max-Min is context-specific and should take into consideration the heterogeneous workload setting.

## 7. Summary of Results and Conclusions

The experimental outcomes revealed the acute need of novel scheduling methods to address the challenges imposed by the heterogeneity of tasks and resources. The existing schedulers generate failures by assigning resources with inherent capacity limitations to tasks with strict processing requirements.

For reasonably large execution workloads, edge devices run out of battery. The conventional scheduling schemes do not properly tackle the higher battery consumption of computationally intensive tasks. The traditional techniques are not energy-aware, without focusing on the power consumption of the computing machines.

The scheduling policies should consider the tasks’ arrival as an extra parameter, given the experienced poor performance results in our realistic edge–cloud experiments.

As discussed, the Round-Robin and SJF routines allocating heterogeneous resources to tasks suffer dramatically from performance degradation.

### Future Work

Having in mind the promising results obtained by the Min-Min and Max-Min algorithms, it is worth creating hybrid implementations of them in order to eliminate task failures. In future work, we plan to build such implementations and investigate their efficiency in heterogeneous contexts, and consequently model additional scheduling constraints based on various real-life use cases.

Furthermore, we plan to also consider dynamic scheduling models, where task characteristics and workload size are not known a priori and where resources’ availability varies.

## Figures and Tables

**Table 1:** Types of read tasks and their processing requirements.

Task Type	Task Length (MI)	Data Size (GB)	Requirements Description
RT1	2,000,000	5	CPU-intensive, memory-intensive
RT2	4,000,000	0.2	CPU-intensive, memory-light
RT3	200,000	5	CPU-light, memory-intensive
RT4	500,000	0.5	CPU-light, memory-light

**Table 2:** Types of write tasks and their processing requirements.

Task Type	Task Length (MI)	Data Size (GB)	Requirements Description
WT1	2,000,000	2	CPU-intensive, I/O-intensive
WT2	1,000,000	0.5	CPU-intensive, I/O-light
WT3	500,000	5	CPU-light, I/O-intensive
WT4	200,000	0.2	CPU-light, I/O-light

**Table 3:** Edge and cloud resource configurations.

Resource Type	Datacenter	Machine	CPU Rating (MI/s)	Memory (GB)	Bandwidth (MB/s)
R	Edge	Raspberry Pi	80,000	1	5
M	Edge	Smartphone	400,000	4	20
C	Cloud	Host	1,000,000	32	80

**Table 4:** Expected execution times of read and write tasks on edge and cloud resources.

Task Type	Execution Time on Resource R (s)	Execution Time on Resource M (s)	Execution Time on Resource C (s)
RT1	-	-	64.50
RT2	90.00	20.00	6.50
RT3	-	-	62.70
RT4	106.25	26.25	6.75
WT1	425.00	105.00	27.00
WT2	112.50	27.50	7.25
WT3	1006.25	251.25	63.00
WT4	42.50	10.50	2.70

**Table 5:** Battery consumption rates of edge devices.

Edge Device	Battery Capacity (mAh)	Processing Drainage Rate (mA)	Data Transfer Drainage Rate (mA)
Smartphone	3600	720	450
Raspberry Pi	2400	800	480

**Table 6:** Experiment parameters.

Entities	Instance Count
Tasks	
Experiment 1	2000
Experiment 2	8000
Resources	
Smartphone	10
Raspberry Pi	5
Cloud	5

**Table 7:** Mean turnaround and waiting time (seconds).

Experiment	Mean Turnaround Time (s)				Mean Waiting Time (s)			
	RR	SJF	Min-Min	Max-Min	RR	SJF	Min-Min	Max-Min
2000 tasks	3006.34	3353.92	1855.41	1851.20	2915.79	3267.13	1807.59	1802.34
8000 tasks	8781.55	8625.32	7170.88	6806.89	8716.57	8566.00	7124.35	6748.55

**Table 8:** Makespan (seconds).

Experiment	RR	SJF	Min-Min	Max-Min
2000 tasks	14,082.50	13,542.50	3783.11	3713.25
8000 tasks	22,434.50	22,025.00	14,236.85	13,941.25

**Table 9:** Throughput (tasks per second).

Experiment	RR	SJF	Min-Min	Max-Min
2000 tasks	0.11	0.12	0.40	0.41
8000 tasks	0.23	0.26	0.42	0.34

## Data Availability

Not applicable.

## Abbreviations

The following abbreviations are used in this manuscript:

BoT	Bag-of-Tasks
CPU	Central Processing Unit
MI	Million Instructions
VM	Virtual Machine
VD	Virtual Device
FIFO	First-In First-Out
IoT	Internet of Things
RR	Round-Robin
SJF	Shortest Job First
